# Aortic valve replacement after coronary artery bypass grafting with the in situ right gastroepiploic artery to the occluded right coronary artery using a temporary vein graft for cardioplegia

**DOI:** 10.1186/s40792-017-0331-1

**Published:** 2017-04-24

**Authors:** Yoshifumi Fuke, Toru Yasutsune, Masato Sakamoto

**Affiliations:** Department of Cardiovascular Surgery, Kitakyushu Municipal Medical Center, Kyushu University, Sakemi 141-11, Okawa city, Fukuoka Japan

**Keywords:** Aortic valve surgery after previous coronary artery bypass grafting, In situ living grafts, Temporary bypass, Cargioplegia

## Abstract

**Background:**

The operation of aortic valve replacement (AVR) after CABG is a technically challenging procedure in respect to dissection of living grafts from its surrounding tissue, myocardial protection, and so on, especially that procedure to patients with living in situ functional arterial grafts to occluded native coronary arteries has a special problem in regard to myocardial protection because myocardial blood supply originates from various arteries including the left internal thoracic artery (LITA), the right internal thoracic artery (RITA), and the right gastroepiploic artery (GEA); hence, adequate myocardial protection should be fastidiously considered.

**Case presentation:**

A 68-year-old woman, who underwent CABG comprised of the in situ LITA to the LAD, the in situ GEA to the RCA, and the saphenous vein graft (SVG) to the obtuse marginal branch of the left circumflex artery (LCX) to the triple vessel coronary disease 9 years before, was referred to our hospital due to the aortic valve stenosis.

**Conclusion:**

We successfully underwent an aortic valve operation to a patient with a functioning LITA to the occluded left anterior descending artery and a GEA to the right coronary artery (RCA) by using a temporary vein graft to the RCA for the infusion of cardioplegic solution in addition to the conventional antegrade and retrograde cardioplegic infusions with ice slush topical cooling.

## Background

Open-heart surgeries after CABG pose on cardiac surgeons difficult problems of the re-sternotomy and dissection of adhesion without injury, myocardial protection, and so on. A functioning arterial graft to the right coronary artery (RCA) is especially troublesome in terms of myocardial protection to the right ventricular (RV) muscle, whereas the conventional antegrade and retrograde cardioplegia infusion might provide enough protection to the left ventricle (LV). We report here a case of successful aortic valve replacement (AVR) after the CABG operation with multiple living arterial in situ grafts to the occluded left anterior descending artery (LAD) and the occluded RCA, using a temporary vein graft for myocardial protection in addition of topical cooling and the conventional antegrade and retrograde cardioplegia.

## Case presentation

**Fig. 1:**
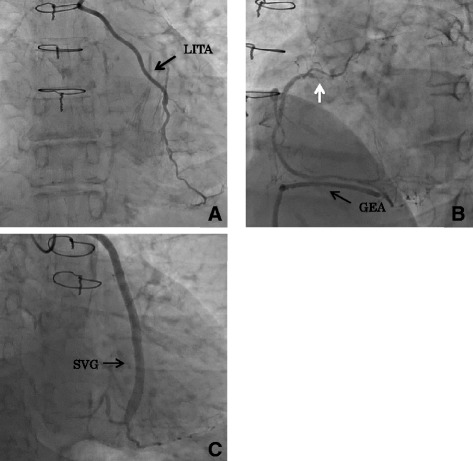
Preoperative coronary angiogram. **a** The in situ LITA to the occluded LAD. **b** The in situ GEA to the occluded GEA. The *arrow* indicates the occlusion of the orifice of the right coronary artery. **c** The saphenous graft between the ascending aorta and the posterolateral branch of the left circumflex artery

A 68-year-old woman with a long history of diabetes mellitus and hypertension, who underwent CABG comprised of the in situ left internal thoracic artery (LITA) to the LAD, the in situ GEA to the RCA, and the saphenous vein graft (SVG) to the obtuse marginal branch of the left circumflex artery (LCX) to the triple vessel coronary disease 9 years before, was referred to our hospital due to the aortic valve stenosis. She suffered a chest pain on exertion 2 months before the presentation. A transthoracic echocardiography showed severe aortic valve stenosis with the aortic valve area (AVA) of 0.83 cm^2^ and the mean transvalvular pressure gradient of 38 mmHg. Her coronary angiography revealed severe triple vessel disease with occlusions of the proximal parts of the LAD and that of the RCA, and all of the bypass grafts were patent (Fig. 1).

At the surgery, a standard re-sternotomy was performed paying careful attention to avoid any injury to mediastinal structures. The living grafts including LITA-LAD, GEA-posterior descending artery, and SVG-LCX were dissected completely freely from adhesion with surrounding tissue, and the adhesion of the heart was completely freed from the pericardium. A cardiopulmonary bypass (CPB) was established by the cannulation to the ascending aorta and to the superior and inferior vena cava respectively, and the left ventricular venting tube was inserted from the right upper pulmonary vein. First and foremost, a new SVG was anastomosed to the mid-portion of the RCA. The ascending aorta was cross-clamped, and the in situ grafts of LITA and GEA were also clamped simultaneously. The initial cardioplegia was infused antegradely from the aortic root and the SVG anastomosed to the RCA, after which the retrograde cardioplegia infusion from the coronary sinus was followed additionally. Topical cooling with ice slush was employed for myocardial protection as well. The ascending aorta was incised obliquely below the proximal anastomosis of the previous SVG to the LCX. The aortic valve was exposed and all three cusps were thickened and densely calcified; their mobility was almost completely lost and the valve area was strictly restricted. The aortic valve was excised and replaced with a 19-mm bioprosthesis. During these procedures, the second cardioplegia was infused from the left coronary orifice, the orifice of the SVG to LCX and the temporary SVG to the RCA antegradely, after which the retrograde cardioplegia was followed as well. The aortotomy was closed, and the clamps of the ascending aorta and living in situ grafts were released under deairing from the LV vent and the aortic root. Once normal sinus rhythm was restored, the patient could be weaned off CPB without much difficulty. The SVG to the RCA was tied off. The durations of the operation, CPB, and the aortic clamp time were 346, 164, and 88 min, respectively. The creatine kinase-MB level on the first postoperative day rose up to 263.4 ng/ml, but a new asynergy of left ventricular wall motion was not recognized. The postoperative transthoracic echocardiography showed the prosthetic valve functioning well with the AVA of 2.06 cm^2^ and the transvalvular peak pressure gradient of 15.3 mmHg. The patient went along with an uneventful recovery course and was discharged on the 15 postoperative days. She was reviewed at our outpatient department 1 month after the discharge and found to be asymptomatic.

## Conclusions

Second cardiac operations after CABG operation are not uncommon nowadays. We have been able to find various reports of open-heart surgeries after CABG articulating some tips of myocardial protection or deadly complications of injury to living grafts [1–3]. When it comes to myocardial protection in aortic valve surgery after CABG, several procedures have been reported; contemporaneous occlusion of the functioning in situ arterial graft and ascending aorta and subsequent cardioplegic arrest as the most common strategy [4], aortic cross-clamp with antegrade and retrograde cardioplegia remaining functional arterial grafts open without dissection as “no-dissection technique” [5, 6], and even beating heart AVR [7]. We reported here a case of AVR with the functioning LITA to the occluded LAD and the GEA to the occluded RCA, where an aortic valve surgery under aortic cross clamp with the conventional combination of the antegrade and retrograde cardioplegia might fail to protect RV muscle due to an anatomical characteristic of the cardiac vein drainage system.

According to the postmortem study of 280 hearts by Ludinghausen [8], only 21 to 25% of all hearts have a small cardiac vein, which enters into the coronary sinus near its estuary, whereas in the heart without a small cardiac vein the blood from RV muscle is drained directly into the RV through Thebesian veins. Retrograde cardioplegia using a coronary sinus catheter, therefore, cannot perfuse cardioplegic solution to the RV muscle properly or provide it with myocardial protective effect adequately. In our case which RV muscle is completely fed by the GEA to the RCA in a physiological condition, the combination of the antegrade and retrograde cardioplegia could not protect that muscle sufficiently under the condition of ascending aorta cross clamping and transient occlusion of the functioning grafts of the LITA and the GEA. Thus, we contrived an antegrade infusion of cardioplegic solution using a transient SVG to RCA in addition to the conventional combination of the antegrade and retrograde cardioplegia to make RV muscle protection more complete.

Another problem of this surgery is dissection of the adhesion of the heart with its pericardium and that of the functioning grafts with surrounding tissue. Impairment of living grafts during the re-sternotomy or the dissection of adhesions might result in lethal complications [2, 3] such as myocardial infarction, perioperative circulatory insufficiency, or postoperative low-output syndrome. A low-output electrocautery is very effective in dissention of adhesion. We could dissect completely the adhesion of the heart with its pericardium and all length of functioning grafts in an appropriate time without any injury. The entire dissection of the heart adhesion with its pericardium could make the effects of topical cooling completely, which is another key factor of myocardial protection than cardioplegia and might play a considerable role in our success in this operation.

As other procedures, an AVR with the “no-dissection technique” [5, 6] and beating heart AVR [7] have been reported. The no-dissection technique is the procedure where functioning grafts are not necessary to dissect or not occluded and is remained open with moderate-to-deep hypothermia during aortic cross-clamping and the cardioplegic arrest under the combined antegrade and retrograde cardioplegia. This procedure might have some disadvantages such as the temperature disparity between circulatory blood supplied through functioning grafts and cardioplegic solution supplied antegradely or retrogradely, the wash-out effect of cardioplegia by blood perfused through functioning grafts, and the impairment of operative vision by blood backflow from coronary artery orifices. The authors concluded that this procedure had led to the non-inferior results from the point of view of mortality and exuding cardiac enzyme, but they did report considerably high incidence of the peri- and postoperative intra-aortic balloon pumping (IABP) usage. These results might suggest the no-dissection technique is useful for some patients but harmful for some patients due to anatomical reasons. Batteleni et al. reported “beating heart AVR” [7], where the heart is beating under the perfusion from coronary sinus retrogradely and from functioning grafts left open during the aortic cross-clamping. We do not have such an experience but are worried about the impairment of operative vision by the backflow from the coronary orifices. For these reasons, we have concluded that to the patients with living grafts after CABG, the AVR under the condition of the clamp of functioning grafts and ascending aorta and the reliable infusion of cardioplegic solution is more appropriate procedure in respect of myocardial protection and the acquisition of the bloodless operative field.

The serum CK-MB level on the first operative day rose up to more than 200 ng/ml despite of sufficient myocardial protection and topical cooling; normally this value of CK-MB should be categorized as a mark of perioperative myocardial infarction. This case had suffered from the severe three-vessel disease occluded the RCA and LAD, and most part of her myocardium was supplied blood from bypass grafts. Retrograde cardioplegic solution from coronary sinus flows through venule, capillary, arteriole and finally coronary artery in anatomical sequence and coronary arteries function as venting vessels during retrograde cardioplegia. The area perfused by LITA and GEA with totally occluded LAD and RCA, hence, loses its venting route during retrograde cardioplegia by clamp of LITA and GEA. This loss of venting route might result insufficient myocardial protection and the moderate increase of CK-MB leakage, which we believe is different from a transmural myocardial infarction so called “ perioperative myocardial infarction” caused by insufficient myocardial protection in case with a normal coronary tree. This hypothesis is supported by the facts of no existence of left ventricular wall motion abnormality in her postoperative echocardiogram, her uneventful postoperative course and unchanged postoperative electrocardiograms compared to her preoperative one despite of her considerable high value of CK-MB. This situation might be a limit of myocardial protection through retrograde cardiplegia to patients with severe lesions of left coronary arteries.

The history of CABG operations is counted as one of the indication criteria for transcatheter aortic valve replacement (TAVR). Our case was a symptomatic 68-year-old female patient with aortic valve stenosis. Considering her expected life longevity and limited durability of valves used in TAVR procedure, surgical AVR, we believe should be indicated in case of this patient. Meticulous preoperative examinations and fastidious operative plan might lead to our desirable result.

We have successfully accomplished an AVR to a patient of aortic valve stenosis after CABG with living in situ arterial grafts to occlude the RCA and the LAD with sufficient topical cooling, and the myocardial protection combined the conventional antegrade, retrograde cardioplegia and the antegrade cardioplegia infusion through the temporary vein graft to the RCA.

## Abbreviations

AVA: Aortic valve area
AVR: Aortic valve replacement
CABG: Coronary artery bypass grafting
CPB: Cardiopulmonary bypass
GEA: Gastroepiploic artery
IABP: Intra-aortic balloon pumping
LAD: Left anterior descending artery
LCX: Left circumflex artery
LITA: Left internal thoracic artery
LV: Left ventricular
RITA: Right internal thoracic artery
RV: Right ventricular
SVG: Saphenous vein graft
TAVR: Transcatheter aortic valve replacement

## References

[1] Mills NL, Everson CT, Hockmuth DR (1991). Technical consideration for myocardial protection during the course of coronary artery bypass reoperation: the impact of functioning saphenous vein and internal mammary artery grafts. J Card Surg.

[2] Gillinov AM, Casselman FP, Lytle BW, Blackstone EH, ParsinsEM LFD (1999). Injury to a patent left internal thoracic artery graft at coronary reoperation. Ann Thorac Surg.

[3] Ivert TS, Ekestrom S, Peterffy A, Welti R (1988). Coronary artery reoperation. Early and late results in 101 patients. Scand J Thorac Cardiovasc Surg.

[4] Odell JA, Mullany CJ, Schaff HV, Orszulak TA, Daly RC, Morris JJ (1996). Aortic valve replacement after previous coronary artery bypass grafting. Ann Thorac Surg.

[5] Byrne JG, Karavas AN, Filsoufi F, Mihaljevic T, Aklog L, Adams DH (2002). Aortic valve surgery after previous coronary artery bypass grafting with functioning intenal mammary artery grafts. Ann Thorac Surg.

[6] Smith RL, Ellman PI, Thompson PW, Girotti ME, Mettler BA, Ailawadi G (2009). Do you need to clamp a patent left internal thoracic artery-left anterior descending graft in reopertaive cardiac surgery?. Ann Thorac Surg.

[7] Battellini R, Rastan AJ, Fabricius A, Moscoso-Luduena M, Lachmann N, Mohr FW (2007). Beating heart aortic valve replacement after previous coronary artery bypass surgery with a patent internal mammary artery graft. Ann Thorac Surg.

[8] Lindinghausen M (1986). Nomenclature and distribution pattern of cardiac vein in man. Proceedings of the second international symposium on myocardial protection via the coronary sinus.

